# EXPLORING THE IMPACT OF ATTENDANCE IN AN OUTDOOR WALKING PROGRAM ON HEALTH IN OLDER ADULTS WITH MOBILITY LIMITATIONS

**DOI:** 10.1093/geroni/igae098.2807

**Published:** 2024-12-31

**Authors:** Tai-Te Su, Ruth Barclay, Rahim Moineddin, Nancy Salbach

**Affiliations:** University of Toronto, Toronto, Ontario, Canada; University of Manitoba, Winnipeg, Manitoba, Canada; University of Toronto, Toronto, Ontario, Canada; University of Toronto, Toronto, Ontario, Canada

## Abstract

Walking in an outdoor environment offers numerous health benefits for older adults with mobility limitations. Effects may vary, however, depending on the level of attendance. This study aimed to investigate whether a dose-response relationship existed between attendance in an outdoor walk group (OWG) program and improvement in health outcomes among older adults with mobility limitations. We analyzed data from a randomized controlled trial conducted across four cities in Canada and focused specifically on community-dwelling older adults assigned to a 10-week OWG program (n=79, age=74.7±6.6 years, 72% female). Participants were categorized into three groups based on attendance tertiles, with the first, second, and third tertiles corresponding to attending 0–9, 10–15, and 16–20 outdoor walking sessions in total. Outcomes were operationalized as change in scores on measures of walking endurance, comfortable and fast gait speed, balance, lower extremity strength, walking self-confidence, and emotional well-being pre- to post-intervention. Results from multiple linear regression models showed that, compared to those who attended 0–9 OWG sessions, participants who attended 16–20 sessions exhibited a 52.7-meter greater improvement in walking endurance (95% CI:12.3, 93.1); 0.15-meter/second greater improvement in comfortable gait speed (95% CI:0.00, 0.29); and 0.17-meter/second greater improvement in fast gait speed (95% CI:0.02, 0.33), after adjusting for sex and study site. No significant dose-response relationships for balance, lower extremity strength, walking self-confidence, and emotional well-being were observed. The role of attendance highlighted in this study provides critical information relevant to the design and implementation of outdoor walking interventions aimed at improving health among older adults.

